# Raftlin is recruited by neuropilin-1 to the activated VEGFR2 complex to control proangiogenic signaling

**DOI:** 10.1007/s10456-020-09715-z

**Published:** 2020-04-09

**Authors:** Asha L. Bayliss, Ananthalakshmy Sundararaman, Camille Granet, Harry Mellor

**Affiliations:** School of Biochemistry, Biomedical Sciences Building, University of Bristol, University Walk, Bristol, BS8 1TD UK

**Keywords:** VEGF receptor, Angiogenesis, Neuropilin, Endothelial cell, Intracellular trafficking

## Abstract

**Background:**

VEGFR2 (vascular endothelial growth factor receptor 2) is the major pro-angiogenic receptor in endothelial cells. Compared to other members of the receptor tyrosine kinase family, we know relatively few VEGFR2 signaling partners. Our objective was to use mass spectrometry-based proteomics to identify novel binding partners of activated VEGFR2.

**Methods:**

We created an endothelial cell line stably expressing GFP-tagged VEGFR2 and isolated activated receptor complexes. Analysis by mass spectrometry identified raftlin as a novel binding partner of VEGFR2.

**Results:**

We found that raftlin is recruited to the activated VEGFR2 complex via the co-receptor Nrp1 (neuropilin-1). We show that raftlin regulates the surface levels of Nrp1 in endothelial cells, controlling the availability of Nrp1 for VEGFR2 interaction. Raftlin stabilizes active VEGFR2 at the cell surface by inhibiting endocytosis of the activated receptor. Raftlin also promotes recycling of internalized VEGFR2 to the cell surface. Raftlin alters the signaling outcomes of VEGFR2 activation, inhibiting the activation of p38 and FAK (focal adhesion kinases) specifically. Both pathways are linked to cell migration in endothelial cells, and raftlin inhibits endothelial cell migration in response to VEGF.

**Conclusion:**

Nrp1 is an important co-receptor for VEGFR2; however, its functions are still only partially understood. We show that raftlin works with Nrp1 in endothelial cells to control intracellular trafficking of the activated VEGFR2. This modulates the response to VEGF and controls endothelial cell migration.

**Electronic supplementary material:**

The online version of this article (10.1007/s10456-020-09715-z) contains supplementary material, which is available to authorized users.

## Introduction

Endothelial cells (ECs) must respond to pro-angiogenic signals to exit their quiescent state and engage in the complex set of changes required for the formation of a new blood vessel [1]. The major pro-angiogenic signal is vascular endothelial growth factor (VEGF), which is secreted by ischemic tissues to drive neovascularization. The major pro-angiogenic receptor on ECs is VEGF receptor 2 (VEGFR2). Activation of VEGFR2 drives a number of downstream signaling pathways that promote the proliferation and survival of ECs as well as controlling the complex set of morphological changes and coordinated migrations that underpin the formation of a new vessel [2].

VEGFR2 is a member of the receptor tyrosine kinase family, which includes a number of related mitogenic and motogenic receptors. The best characterized of these is epidermal growth factor receptor 1 (EGFR). EGFR signals to a similar set of signaling partners as VEGFR2 to control the proliferation and migration of epidermal cells [3]. Significant effort has been made to map these downstream partners, with the use of quantitative proteomics to identify the components of the active EGFR receptor complex. As a result, we have a detailed picture of EGFR interactome, which is now thought to comprise over 200 proteins [4–6]. Assembling this information has been critical to understanding the complex signaling networks controlled by the receptor, and for identifying targets for drug development.

In contrast, we know a far smaller set of interactions for VEGFR2 and a far smaller number of validated signaling partners [2, 7]. Here, we use proteomic analysis of isolated VEGFR2 complexes to identify novel signaling partners for this receptor. We show that the poorly characterized membrane protein raftlin is recruited to the activated VEGFR2 complex to control EC migration in response to VEGF. This study identifies a novel component of the VEGFR2 complex and demonstrates the importance of proteomic analysis in the development of our understanding of VEGFR2 signaling.

## Methods

### Reagents

Human recombinant VEGF_165_ was from Peprotech, London, UK. Human recombinant VEGF_112_ was from R&D Systems, Minneapolis, MN. A mouse monoclonal antibody to VEGFR2 (Clone No. 89,109) was from R&D Systems and was used for immunofluorescence microscopy and antibody uptake experiments. A rabbit monoclonal antibody to VEGFR2 (Clone No. 55B11) was from Cell Signaling Technologies (London, UK) and was used for western blotting. A goat anti-Nrp1 polyclonal antibody was from Santa Cruz Biotechnology, Dallas, TX. A rabbit polyclonal antibody to raftlin was from Proteintech, Manchester, UK. A mouse monoclonal antibody to EEA1 was from BD Bioscience, Oxford, UK. A rabbit monoclonal antibody to sodium/potassium ATPase (Clone No. D4Y7E) was from Cell Signaling Technologies. Antibodies to phosphorylated VEGFR2 (Tyr1175 and Tyr1059), Erk1/2 (Thr202/Tyr204), Src (Tyr416), (FAK (Tyr397), FAK (Tyr925), PLCγ (Tyr793), and p38 (Thr180/Tyr182) were from Cell Signaling Technologies. Antibodies to phosphorylated FAK Tyr407 were from ThermoFisher Scientific, Loughborough, UK. Antibodies to phosphorylated VEGFR2 (Tyr1214) were from R&D Systems. siRNA oligonucleotides targeting raftlin and Nrp1 were from Dharmacon, Lafayette, CO and had the following sequences: siRFTN-A, GAAAUUCGUUGGUGUUAUA; siRTN-B, GAGAAGAAAUGCACAACAG; siNrp1, CCAGAGAAUUAUGAUCA. A universal negative control siRNA oligonucleotide was from Sigma-Aldrich, Poole, UK. A plasmid encoding full-length human raftlin was created in the mammalian expression vector pcDNA3 (Takara Bio, Mountain View, CA).

### Cell culture and transfection

Human umbilical vein ECs (HUVEC) from pooled donors were purchased from Lonza, Cambridge, UK and were cultured in complete EC growth media (EGM-2; Lonza). Conditionally immortalized kidney ECs (CiGEnC) were maintained in complete microvascular EC growth media (EGM-2 MV; Lonza) at 33 °C and switched to 37 °C for experimental work. Where indicated, cells were transfected with siRNA oligonucleotides using GeneFECTOR lipid according to the manufacturer’s protocol (Venn Nova, Pompano Beach, FL) and used 48 h later. Transfections with expression vectors were performed using Lipofectamine 2000 according to the manufacturer’s protocol (ThermoFisher Scientific).

### Creation of a stable GFP-VEGFR2 endothelial cell line

Human VEGFR2 cDNA was modified to replace the signal peptide with a strong IgG kappa signal peptide followed by enhanced GFP. The resultant mature protein has GFP fused at the natural VEGFR2 N-terminus. This construct was subcloned into the pLVXpuro vector (Takara Bio). Lentiviral particles were produced in a HEK293T packaging line, using standard methods. Briefly, cells were transfected with pLVXpuro and the packaging vectors pMDG2 and psAX2, using polyethylenimine. After 48 h, the cell medium was added to CiGEnC cells overnight. The following day, the process was repeated with fresh virus. Cells were selected for stable expression in media supplemented with 2 µg/ml puromycin.

### Isolation of VEGFR2 complexes and mass spectrometry

CiGEnCs stably expressing GFP-VEGFR2 or GFP were serum-starved for 1 h and then stimulated with 40 ng/ml VEGF for the indicated time points. The cells were then washed once with ice-cold PBS and harvested in lysis buffer (20 mM Tris pH7.5, 137 mM NaCl, 0.5% NP-40, 1 mM EDTA, 2 mM sodium orthovanadate, 10 mM sodium fluoride) containing protease inhibitor cocktail (Sigma-Aldrich). Cell lysates were clarified by centrifugation for 12 min at 12,000 × *g* and 4 °C. The lysates were then incubated with GFP-Trap agarose beads (ChromoTek, Martinsried, Germany) for 1 h with end-over-end rotation at 4 °C. The beads were washed three times with lysis buffer and the bound protein eluted by heating at 95 °C with SDS-PAGE sample buffer. Samples were run approximately 1 cm into the separating gel of an SDS-PAGE gel and then subjected to in-gel tryptic digestion. The resulting peptides were fractionated using an Ultimate 3000 nanoHPLC system in line with an LTQ-Orbitrap Velos mass spectrometer (ThermoFisher Scientific). The raw data files were processed and quantified using Proteome Discoverer software v1.4 (ThermoFisher Scientific) and searched against the UniProt Human database using the SEQUEST algorithm.

### Immunofluorescence microscopy

Cells were prepared for confocal immunofluorescence microscopy by fixation in 4% paraformaldehyde. Confocal microscopy was performed using a Leica SP5 AOBS confocal laser-scanning microscope with an attached Leica DM I6000 inverted microscope. Confocal sections were taken across the z-plane and processed to form a 2D projection representing the full depth of the cell culture.

### Immunoprecipitation

HUVEC were serum-starved for 1 h followed by stimulation with 40 ng/ml VEGF for the indicated time points. Cells were then washed once with ice-cold PBS and harvested in lysis buffer (20 mM Tris pH7.5, 137 mM NaCl, 0.5% NP-40, 1 mM EDTA, 2 mM sodium orthovanadate, 10 mM sodium fluoride) containing protease inhibitor cocktail (Sigma-Aldrich). Cell lysates were clarified by centrifugation for 12 min at 12,000 × *g* and 4 °C. A sample was taken from the supernatant, which represented the input, and the remainder was added to VEGFR2, raftlin or IgG control antibody, as indicated. Following 30 min of end-over-end rotation at 4 °C, lysates were incubated with Protein G beads (Sigma-Aldrich; 10 µL packed beads per 500 µL of lysate) for a further 2 h with end-over-end rotation at 4 °C. The beads were then washed three times in lysis buffer at 4 °C and protein was extracted from the beads by heating at 95 °C with SDS-PAGE sample buffer. Equivalent volumes of all samples were resolved by SDS-PAGE and analyzed by western blotting.

### Antibody uptake

HUVEC seeded on glass coverslips were serum-starved for 1 h, washed once with ice-cold basal EGM-2, and incubated with VEGFR2 antibody for 25 min on ice. Cells were washed twice with ice-cold basal media followed by incubation with VEGF at 37 °C. At the indicated time point, cells were washed once with ice-cold PBS and fixed with 4% paraformaldehyde for 15 min at room temperature. Standard immunofluorescence staining procedure was then followed and images were taken on a confocal microscope, as described above. Vesicles were counted using the ‘Trackmate’ function of Image J.

### Biotinylation

HUVEC were serum starved and stimulated with VEGF where indicated. All following steps were performed at 4 °C. Cells were washed with complete PBS (Sigma-Aldrich) and incubated 0.2 mg/ml NHS-Biotin (ThermoFisher Scientific) in complete PBS for 30 min. The unreacted biotinylation reagent was quenched by incubation with 100 mM glycine. Cells were then solubilized in lysis buffer (20 mM Tris pH 7.5, 137 mM NaCl, 1 mM EDTA, 1% NP40, 2 mM sodium orthovanadate, 10 mM sodium fluoride) containing protease inhibitor cocktail (Sigma-Aldrich). Cell lysates were centrifuged at 12,000 × *g* for 12 min at 4 °C and a sample was taken from the supernatant, which represented the total cellular protein. Streptavidin-agarose beads (Upstate Biotechnology, Lake Placid, NY) were added to the remaining supernatant (20 µl packed beads per 500 µl lysate) and left to tumble at 4 °C for 1 h. Beads were collected by centrifugation at 800 × *g* for 30 s at 4 °C and supernatant was removed—this sample represents the internal protein pool. The beads were then washed 3 times in lysis buffer at 4 °C and protein was extracted from the beads by heating at 95 °C with SDS-PAGE sample buffer—this represents the surface pool. Equivalent volumes of all three samples were resolved by SDS-PAGE and analyzed by western blotting.

### Cell fractionation and ultracentrifugation

Two 10 cm dishes of HUVEC per condition were treated with control or raftlin siRNA. After 48 h, cells were serum-starved and stimulated with 40 ng/ml VEGF for 30 min at 37 °C. Cells were washed once with ice-cold PBS and once with ice-cold homogenizing buffer (HB; 250 mM sucrose, 8 mM CaCl_2_, 4 mM MgCl_2_, 78 mM KCl, 10 mM EGTA, 5 mM HEPES, pH 7.2) containing protease inhibitor cocktail (Sigma-Aldrich), followed by the addition of 1.1 ml of HB buffer per dish. The remainder of the steps were carried out on ice. Cells were scraped, collected into a 1.5 ml microcentrifuge tube, and passed ten times through an Isobiotec cell homogenizer containing a tungsten carbide ball bearing that gives a 10 µm clearance (Isobiotec, Heidelberg, Germany). Homogenates were centrifuged for 5 min at 1000 × *g* and 4 °C. A sample of the supernatant was added to SDS-PAGE sample buffer to represent the input sample. 2 ml homogenate was then placed on top of a 10–30% iodixanol gradient in an ultracentrifuge tube. After centrifugation at 24,700 × *g* for 18 h at 4 °C, 0.5 ml fractions were collected from the top of the tube and added to SDS-PAGE sample buffer. Equal volumes were resolved by SDS-PAGE and analyzed by western blotting.

### Signaling assays

HUVEC were serum-starved, stimulated with VEGF for the indicated time points and then harvested in lysis buffer (20 mM Tris pH 7.5, 137 mM NaCl, 1% NP-40, 1 mM EDTA, 2 mM sodium orthovanadate, 10 mM sodium fluoride) containing protease inhibitor cocktail (Sigma-Aldrich). Cell lysates were clarified by centrifugation for 12 min at 12,000 × *g* and 4 °C. Lysates were then added to SDS-PAGE sample buffer and heated at 95 °C. Equivalent volumes were resolved by SDS-PAGE and analyzed by western blotting.

### Cell migration assays

For analysis of cell motility, HUVEC were transfected with control or raftlin siRNA. After 48 h, cells were seeded at low density to allow the observation of single cells. Cell motility was tracked over 14 h using an Incucyte ZOOM system (Essen Bioscience, Welwyn Garden City, UK). Cell velocity and directness of migration were calculated using Incucyte ZOOM software. For analysis of cell migration, HUVEC were transfected with control or raftlin siRNA. After 24 h, cells were seeded into a 96-well ImageLock plate (Essen Bioscience) and incubated for a further 24 h until confluent. Cells were serum-starved for 1 h and stimulated with 40 ng/ml VEGF. A scratch was made in each well using the IncuCyte WoundMaker tool (Essen Bioscience) and the plate was installed in the IncuCyte ZOOM system. Images were taken at 10 × magnification every hour for 24 h. The percentage of wound closure was calculated using the Incucyte ZOOM software.

### Statistical analysis

Statistical significance was determined using two-way ANOVA with a post-hoc Bonferroni multiple comparison test. * *P* < 0.05, ** *P* < 0.01, **** *P* < 0.001, **** *P* < 0.0001.

## Results

### Identification of novel binding partners of activated VEGFR2

**Fig. 1:**
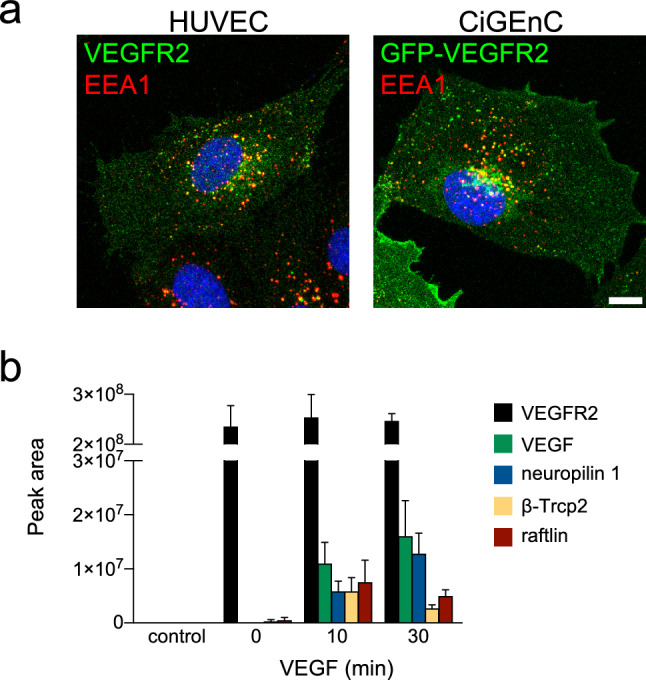
Identification of novel VEGFR2 binding partners. **a** The localization of endogenous VEGFR2 (green) in HUVEC was compared with that of GFP-VEGFR2 expressed in CiGEnC. The GFP-tagged receptor showed a similar pattern of plasma membrane and vesicular localization. In both cases, VEGFR2 + vesicles co-localized extensively with the early endosomal marker EEA1. Bar = 10 µm. **b** CiGEnC expressing GFP-VEGFR2 were stimulated with 40 ng/ml VEGF and VEGFR2 complexes were captured and analyzed by mass spectrometry. The graph shows the peak areas of five proteins detected in the VEGFR2 complex, a comparative measure of abundance. None of these proteins were detected in immunoprecipitates from a control CiGEnC cell line transduced with GFP only. Binding of VEGF to the receptor could be detected, together with the recruitment of the well-characterized binding partners neuropilin-1 and β-Trcp2. The novel VEGFR2 partner raftlin was identified in VEGFR2 complexes upon VEGF stimulation. Data are means ± SEM; *n* = 3

In order to purify binding partners of VEGFR2, we created a stable endothelial cell line expressing a GFP-tagged VEGFR2 receptor. We constructed a fusion protein whereby the signal peptide of VEGFR2 was replaced with the strong signal peptide from the IgG kappa light chain to promote successful surface expression. This was followed by a GFP cassette so that the mature receptor was tagged at its extracellular N-terminus. This strategy left the intracellular C-terminal domain free to interact with downstream signaling and trafficking partners. We used lentiviral transduction to allow stable expression of the construct in the CiGEnC endothelial cell line. CiGEnC are conditionally immortalized human kidney endothelial cells (ECs) and maintain expression of a wide range of endothelial markers [8]. The distribution of GFP-VEGFR2 in this cell line was essentially identical to that of the endogenous receptor in primary human umbilical vein ECs (HUVEC). Both cell types showed a characteristic vesicular pool that overlapped significantly with the early endosomal marker EEA1 ( [9]; Fig. 1a). The GFP-tagged receptor was activated normally by VEGF stimulation, with phosphorylation of the receptor itself, and of key pro-angiogenic signaling partners (Fig. S1a).

Cells were stimulated with VEGF over a 30-min time course and VEGFR2 complexes were isolated by affinity purification using GFP-Trap agarose beads. The samples were analyzed by mass spectrometry using an LTQ-Orbitrap Velos mass spectrometer and compared to a CiGEnC line expressing GFP alone. The analysis identified VEGFR2 as the strongest hit, together with a number of known VEGFR2 binding partners. The full proteomic datasets from three independent experiments are shown in Table 1 in the online-only Data Supplement. The method was sensitive enough to detect binding of VEGF to the receptor, which remained associated with VEGFR2 across the time course (Fig. 1b). As previously reported, VEGF stimulation promoted the binding of the important VEGFR2 co-receptor; neuropilin-1 (Nrp1). We also saw recruitment of the ubiquitin E3 ligase b-Trcp2 (Fig. 1b), which plays a key role in mediating the degradation of the activated receptor [10, 11]. In addition to previously characterized VEGFR2 partners, we saw consistent recruitment of raftlin to the activated receptor (Fig. 1b). Raftlin was matched to various accession numbers, depending on the run (Q14699, B4E2S3, G3XAJ6; Table 1 in the online-only Data Supplement). Raftlin is a poorly characterized membrane protein that has been shown to play a part in lymphocyte signaling [12, 13]. No previous work links raftlin to VEGFR2 signaling, or to a function in ECs.

### Raftlin is recruited to activated VEGFR2 via Nrp1

**Fig. 2:**
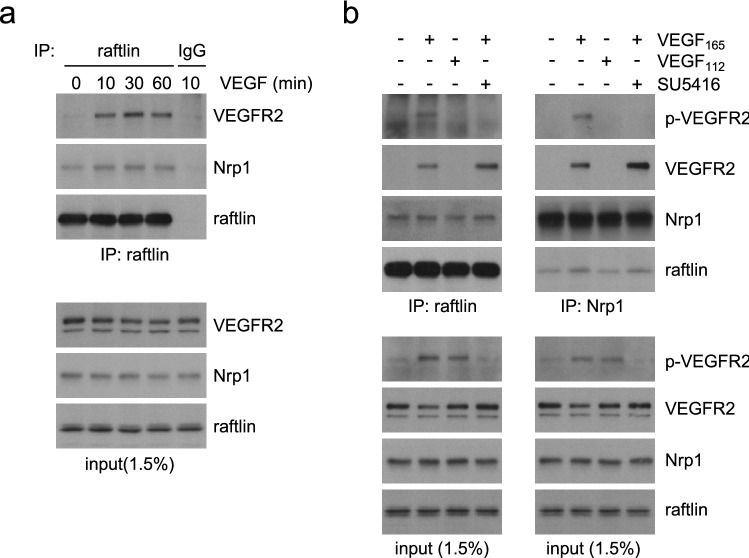
Raftlin is recruited to activated VEGFR2 via Nrp1. **a** HUVEC were stimulated with 40 ng/ml VEGF over a 60-min time course prior to immunoprecipitation of endogenous raftlin. Raftlin formed a pre-complex with Nrp1 and interacted with VEGFR2 upon VEGF stimulation. The control immunoprecipitation was with a non-specific IgG. Total cell lysates contained both the mature VEGFR2 and the lower molecular weight, immature receptor. Only the mature receptor interacted with raftlin. **b** HUVEC were stimulated for 10 min with either 40 ng/ml VEGF165 or VEGF112. Where indicated, cells were pre-incubated with 5 μM SU5416 for 1 h. Endogenous raftlin or Nrp1 was isolated from cell lysates by immunoprecipitation. Nrp1 and raftlin were only recruited to the activated VEGFR2 complex when cells were stimulated with VEGF165. Pre-treatment with SU5416 inhibited VEGFR2 phosphorylation at Y1175 (P-VEGFR2) but did not affect complex formation with Nrp1-raftlin

We validated the interaction between VEGFR2 and raftlin through immunoprecipitation of endogenous raftlin from primary human ECs. Endogenous VEGFR2 co-immunoprecipitated with raftlin upon VEGF stimulation (Fig. 2a). We confirmed the interaction in reverse by immunoprecipitating endogenous VEGFR2 and blotting for raftlin (Fig. S1b). Interestingly, endogenous Nrp1 also co-immunoprecipitated with raftlin and this was independent of VEGF stimulation (Fig. 2a). This suggested that raftlin may be recruited to active VEGFR2 indirectly via Nrp1. To test this, we compared complex formation in ECs stimulated with VEGF_112_. This artificially truncated growth factor is incapable of binding Nrp1 but can still activate VEGFR2 [14]. As previously published [14], VEGF_112_ drove VEGFR2 activation to the same extent as the VEGF_165_ isoform but did not support formation of a VEGFR2-Nrp1 complex (Fig. 2b). Similarly, VEGF_112_ did not support the formation of a VEGFR2-raftlin complex (Fig. 2b). Incubation with the VEGFR2 kinase inhibitor SU5146 completely blocked autophosphorylation of VEGFR2 in response to VEGF_165_ but did not affect complex formation with either Nrp1 or raftlin (Fig. 2b). We conclude that raftlin exists as a pre-complex with Nrp1 in ECs and that this complex is recruited to activated VEGFR2 via the heterodimerization of VEGFR2 and Nrp1. This interaction is independent of VEGFR2 phosphorylation. Raftlin shows 29% homology to the related protein raftlin-2 [13]. We did not detect raftlin-2 peptides by mass spectrometry of VEGFR2 complexes (Table 1 in the online-only Data Supplement) and we were not able to detect raftlin-2 in immunoprecipitates of activated VEGFR2 (Fig. S1c). We conclude that the interaction with VEGFR2 is specific for raftlin.

### Raftlin controls the surface localization of Nrp1

**Fig. 3:**
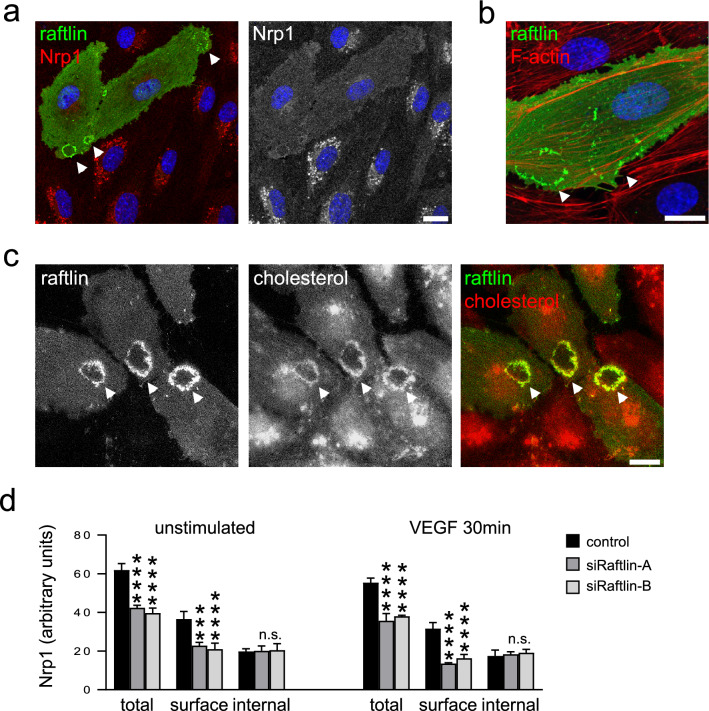
Raftlin controls the surface localization of Nrp1. **a** HUVEC were transiently transfected with raftlin (green) and stained for endogenous Nrp1 (red). Overexpression of raftlin caused loss of the intracellular vesicular pool of Nrp1 and an increase in the diffusely stained surface pool. Raftlin showed diffuse surface staining but was also concentrated in annular structures (arrowheads). **b** HUVEC were transfected with raftlin (green) and co-stained for F-actin (red). The annular raftlin structures do not contain F-actin filaments. **c** HUVEC were transfected with raftlin (green) and co-stained for cholesterol (red). The annular raftlin structures are cholesterol-rich. Bars = 10 µm. **d** HUVEC were transfected with raftlin siRNA or control. Cells were treated with or without 40 ng/ml VEGF for 30 min. Surface proteins were the biotinylated on ice and isolated using streptavidin beads. A representative western blot is shown in Figure ID in the online-only Data Supplement. Silencing of raftlin reduced surface Nrp1 and led to a further loss of surface Nrp1 on VEGF stimulation. Data are means ± SEM (*n* = 3)

Raftlin has previously been reported to localize to cholesterol-rich membrane domains at the surface of lymphocytes via fatty acylation [13]. Given the existence of a raftlin–Nrp1 complex (Fig. 2), we were interested in determining the sites of localization of raftlin and Nrp1 in ECs. The available raftlin antibodies did not have the required sensitivity to detect endogenous raftlin by immunofluorescence microscopy, and so we expressed raftlin in ECs by transient transfection to increase the signal. Endogenous Nrp1 was divided between the plasma membrane and a vesicular pool as previously reported ( [15]; Fig. 3a). Surprisingly, raising the cellular expression of raftlin led to a loss of vesicular Nrp1, and a corresponding increase in the surface pool (Fig. 3a). Raftlin showed a diffuse distribution through the plasma membrane but was also detected in bright annular structures (Fig. 3a). These structures bore superficial resemblance to podosome rosettes and also to circular dorsal ruffles. Podosome rosettes are sites of turnover and remodeling of the extracellular matrix at the basal surface of ECs [16], whereas circular dorsal ruffles are sites of endocytosis at the apical surface [17]. A defining feature of both podosome rosettes and dorsal circular ruffles is their dependence on localized F-actin, which was absent from the raftlin rings (Fig. 3b). Instead, staining with the cholesterol-binding drug filipin showed that these structures were cholesterol-rich (Fig. 3c). These findings suggested that raftlin might act to stabilize the surface pool of Nrp1 through its anchoring to membrane microdomains. To investigate this, we used surface biotinylation to quantify the surface and internal pools of Nrp1 in the presence and absence of raftlin. We selected siRNA oligonucleotides that gave effective silencing of endogenous raftlin in ECs (Fig. S1d). Surface biotinylation was used to label total surface proteins, followed by capture on streptavidin beads. We then used western blotting to quantify the levels of surface (biotinylated) and intracellular (non-biotinylated) Nrp1. Silencing of raftlin led to an approximately 30% decrease in total Nrp1, which was entirely due to a 50% reduction in the surface pool (Fig. 3d, S1e). On treatment with VEGF, the surface pool of Nrp1 remained constant in control cells; however, in cells depleted of raftlin, there was further loss of surface Nrp1 (Fig. 3d). We conclude that raftlin acts to stabilize Nrp1 at the EC surface, maintaining levels in resting cells and preserving surface Nrp1 upon VEGF stimulation.

### Raftlin controls the internalization of VEGFR2

**Fig. 4:**
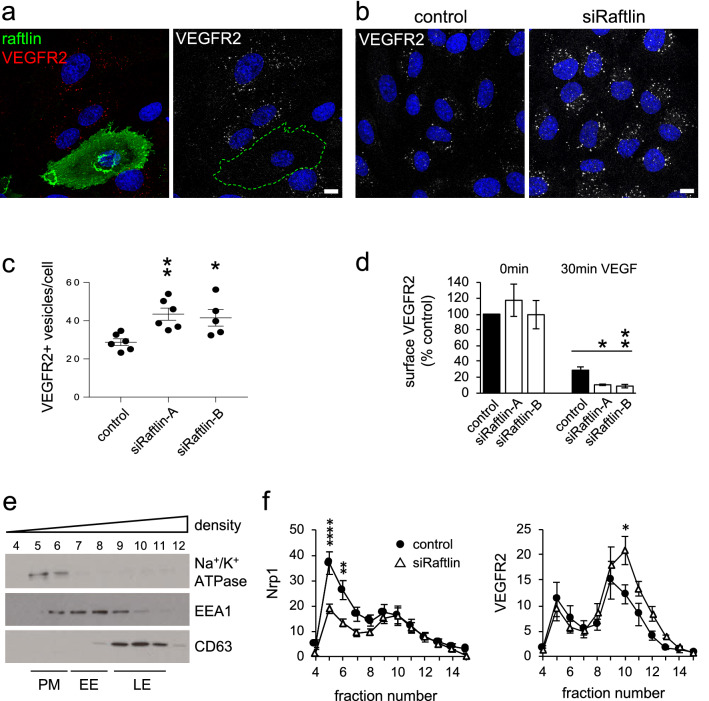
Raftlin controls the internalization of VEGFR2. **a** HUVEC were transfected with untagged full-length raftlin (green) and pre-labeled on ice with VEGFR2 antibody (red). Antibody uptake was measured after 30-min stimulation with 40 ng/ml VEGF. Overexpression of raftlin blocked VEGFR2 internalization into endosomes. Bar = 10 μm. **b**–**c** HUVEC were transfected with raftlin siRNA or control and the same uptake experiment performed. Silencing of raftlin significantly increased the internalization of VEGFR2 into endosomes. Data are means ± SEM (*n* = 3). **d** HUVEC were transfected with raftlin siRNA or control. Cells were treated ± 40 ng/ml VEGF for 30 min. Surface proteins were the biotinylated on ice and isolated using streptavidin beads. Silencing of raftlin had no effect on surface VEGFR2 in unstimulated cells but led to increased internalization on VEGF-stimulation. Data are means ± SEM (*n* = 3). **e** HUVEC cellular membranes were purified by density centrifugation. Western blotting of gradient fractions revealed the positions of the plasma membrane (PM), early endosome (EE), and late endosome (LE) fractions. **f** HUVEC were treated with raftlin siRNA or control and stimulated with 40 ng/ml VEGF for 30 min. The intracellular distributions of Nrp1 and VEGFR2 were determined by western blotting of gradient fractions. Silencing of raftlin caused a loss of surface Nrp1 and an increase of VEGFR2 in late endosomal fractions. Data are means ± SEM (*n* = 4)

Anchoring of Nrp1 at the cell surface by raftlin would potentially inhibit endocytosis of the VEGFR2-Nrp1 complex on activation. To examine this, we labeled surface VEGFR2 in live ECs by incubation with a specific monoclonal antibody and then examined the internalization of the receptor over a 30-min period of VEGF stimulation. In control ECs, VEGFR2 was internalized into a vesicular endosomal pool as expected; however, receptor uptake was completely blocked in cells overexpressing raftlin (Fig. 4a). We repeated the experiment in cells depleted of raftlin to determine the contribution of endogenous raftlin to VEGFR2 endocytosis. Silencing of raftlin caused a significant increase in the number of VEGFR2 + vesicles on VEGF stimulation (Fig. 4b, c). We used surface biotinylation to quantify the effect on the internalization of activated VEGFR2. Depletion of raftlin had no effect on the surface levels of VEGFR2 in resting cells; however, significantly more VEGFR2 was removed from the cell surface on VEGF stimulation (Fig. 4d), consistent with the increased rate of internalization.

Nrp1 has been implicated in the recycling of VEGFR2 back to the cell surface after endocytosis [15]. Given that loss of raftlin perturbed the distribution of Nrp1 between the surface and vesicular compartments (Fig. 3a), we were interested to see what happened to endocytosed VEGFR2 in ECs depleted of raftlin. To address this, we stimulated ECs for 30 min with VEGF and then fractionated cellular membranes by density centrifugation. We used well-characterized markers of cell compartments to identify the fractions corresponding to the plasma membrane, early endosomes, and late endosomes on the gradient (Fig. 4e). In accordance with the surface biotinylation data (Fig. 3d), silencing of raftlin caused a marked loss of plasma membrane Nrp1, without affecting the internal cellular pool (Fig. 4f, S2). In contrast, silencing of raftlin caused accumulation of internalized VEGFR2 in the late endosomal compartment, consistent with an inhibition of receptor recycling (Fig. 4f, S2). We conclude that raftlin acts to inhibit VEGFR2 internalization through formation of a VEGFR2-Nrp1-raftlin complex at the cell surface. Raftlin also acts to promote recycling of internalized VEGFR2 by ensuring the availability of Nrp1 at the cell surface to pair with the receptor in its journey though the cell.

### Raftlin controls VEGFR2 signaling output

The intracellular trafficking of tyrosine kinase receptors plays a key role in shaping their signaling outputs [18]. Internalized activated VEGFR2 continues to signal from inside the cell, and changes to the kinetics of VEGFR2 traffic alter the pattern and kinetics of signals generated by the receptor [19, 20]. Association of VEGFR2 with its co-receptor Nrp1 has also been found to modulate VEGFR2 signaling. Previous studies have shown that Nrp1 generally enhances sensitivity of ECs to VEGF and acts specifically to change the signaling to specific downstream pathways [21]. Given the effects of raftlin on the trafficking of VEGFR2 and on the intracellular distribution of Nrp1, we were interested in determining if these changes affected the signaling output from the receptor.

**Fig. 5:**
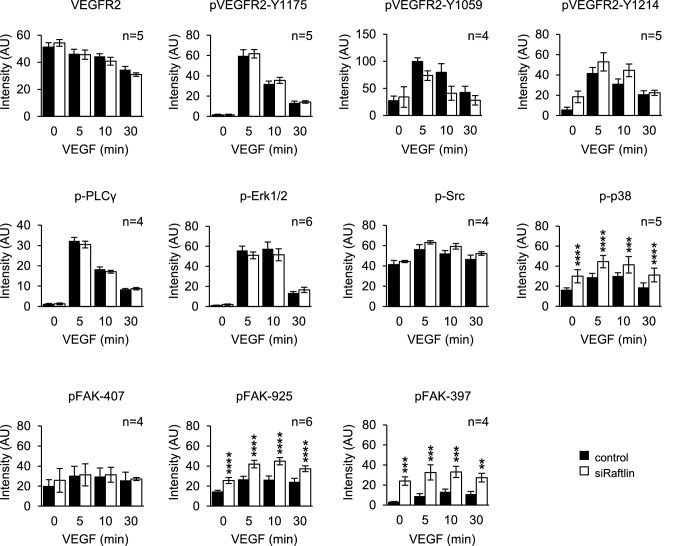
Raftlin regulates VEGFR2 signaling. HUVEC were transfected with raftlin siRNA or control and then stimulated with 40 ng/ml VEGF over a time course of 30 min. The activation of key downstream VEGFR2 signaling partners was quantified by western blotting and densitometry. Depletion of raftlin had no significant effect on VEGFR2 activation, but instead specifically increased the activation of p38 and FAK. Data are means ± SEM from multiple independent experiments, as indicated in the panels

In keeping with previous reports [22], we found that silencing of Nrp1 reduced VEGFR2 phosphorylation at Tyr1175 (Fig. S3). We saw a corresponding general decrease in activation of downstream signaling partners (Fig. S3). Previous work has shown that Nrp1 is specifically required for activation of p38 downstream of VEGFR2 [14]. Accordingly, we found that silencing of Nrp1 abolished activation of this key signaling partner in response to VEGF stimulation (Fig. S3). Silencing of raftlin had no effect on the phosphorylation of Tyr1175 of VEGFR2 in response to VEGF stimulation (Fig. 5). This is a major phosphorylation site on the activated receptor and recruits PLCγ [1]. Consistent with this, we saw no effect of raftlin depletion on the activation of PLCγ or Erk1/2, nor did we see an effect on the activation of Src (Fig. 5). Surprisingly, silencing of raftlin had the opposite effect to silencing of Nrp1 on p38 activation, with a significant increase in p38 phosphorylation in response to VEGF stimulation (Fig. 5). Intriguingly, silencing of raftlin increased the phosphorylation of VEGFR2 at Tyr1214, the site responsible for p38 activation [2], although this trend did not achieve statistical significance (Fig. 5). Previous studies have provided evidence that Nrp1 is required for the phosphorylation of focal adhesion kinase (FAK) downstream of VEGFR2 activation; specifically, at Tyr407 [2]. Those studies used a Nrp1 mutant that was unable to bind VEGFR2 [23]. We found that silencing of Nrp1 did not affect FAK phosphorylation at Tyr407 (Fig. 3). Similarly, silencing of raftlin did not affect phosphorylation of this site either (Fig. 5). We examined the phosphorylation of FAK at two other sites: Tyr397 and Tyr925. Phosphorylation of Tyr397 is a marker of FAK activation [24] and Tyr397 is a major target of phosphorylation downstream of VEGFR2 activation [25]. Tyr925 is a minor site of phosphorylation in FAK downstream of VEGFR2 activation [26]. Interestingly, silencing of either Nrp1 or raftlin led to a significant increase in FAK phosphorylation at both sites (Fig. 5, S3). We conclude that raftlin acts to change the balance of signals produced by VEGFR2, leaving activation of the Src and Erk1/2 pathways unchanged, but suppressing the activation of p38 and FAK.

### Raftlin controls endothelial cell migration

The best-characterized role for Nrp1 in VEGFR2 signaling is in the regulation of EC migration [21]. Several studies have demonstrated that Nrp1 enhances the effects of VEGFR2 activation on motility [14, 23, 27]. There appear to be multiple mechanisms by which Nrp1 can do this. Nrp1 is required for the activation of p38 by VEGFR2 [14] and p38 activation is required for VEGF-induced chemotaxis [28]. There is also evidence to support a requirement Nrp1 in the phosphorylation of p130Cas downstream of VEGFR2, which is also required for chemotaxis [22]. Finally, phosphorylation of FAK at Tyr407 has also been linked to the regulation of EC migration by Nrp1 [2]. We did not see changes to p130Cas phosphorylation in ECs on silencing of either Nrp1 or raftlin (data not shown), and we did not see changes to FAK phosphorylation at Tyr407 (Fig. 5, S3). We did see increased activation of p38 on silencing of raftlin (Fig. 5) and increased activation of FAK (Fig. 5). Importantly, we saw increases in FAK phosphorylation at Tyr925, which is also linked to increased cell migration [29].

**Fig. 6:**
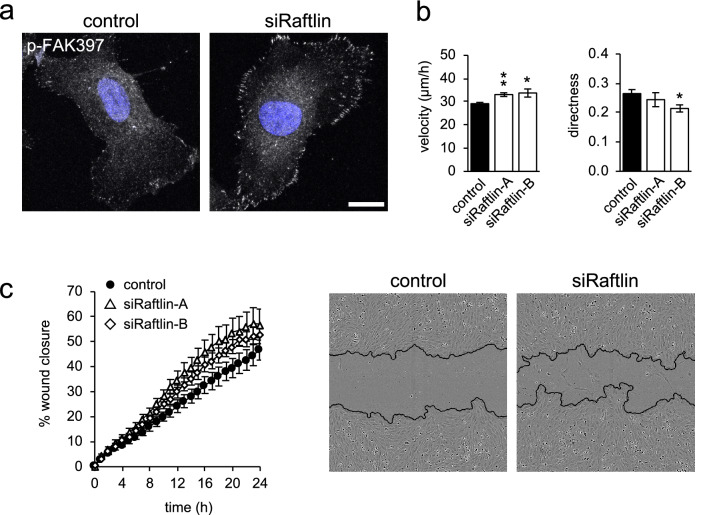
Raftlin regulates EC migration **a** HUVEC were transfected with raftlin siRNA or control and then stimulated with 40 ng/ml VEGF for 5 min. Depletion of raftlin increased the intensity of staining of autophosphorylated FAK (Tyr397) at focal adhesions. Bar = 10 µm. **b** HUVEC were transfected with raftlin siRNA or control and then plated at low density to allow tracking of cell movement over 14 h. The average velocity and directness of cells were quantified. Directness was calculated as the ratio of the Euclidian distance traveled to the total migrated distance, with a value of 1 corresponding to a cell moving in a straight line. Silencing of raftlin increased cell velocity and decreased directness of movement. Data are means ± SEM (*n* = 5). **c** HUVEC were transfected with raftlin siRNA or control and subjected to a scratch migration assay stimulated with 40 ng/ml VEGF. Silencing of raftlin significantly increased cell migration. Data are means ± SEM (*n* = 6). The panels show representative images obtained 16 h post wounding

We examined FAK activation in ECs by immunofluorescence microscopy. Silencing of raftlin led to a dramatic increase in staining for active FAK (Tyr397), which was localized to focal adhesions at the cell periphery (Fig. 6a). Activation of FAK leads to the turnover of these adhesive contacts, promoting cell migration [24]. We measured the motility of ECs by tracking the random movements of single cells. Silencing of raftlin led to an increase in the velocity of movement and a decrease in directness (Fig. 6b). Directness is defined as the ratio between the Euclidian distance traveled (the distance between the start and end point) and the total distance traveled. A decrease in directness shows that cells are moving in a less persistent manner, with increased changes in direction. Small changes in cell velocity and persistence become magnified over distance during chemotaxis. We analyzed the migration of ECs in response to VEGF-stimulation using the well-characterized scratch migration model. Silencing of raftlin significantly increased the migration of ECs in response to VEGF (Fig. 6c). We conclude that raftlin acts to modulate the VEGFR2 signaling response by suppressing cell migration, while leaving activation of the proliferative Erk1/2 pathway untouched.

## Discussion

Here, we show that raftlin is a novel regulator of VEGFR2, controlling both its intracellular trafficking and signaling output. Raftlin was first identified as a major component of lipid rafts in B-cells, where it regulates signaling from the B-cell receptor [13]. In ECs, lipid rafts facilitate VEGFR2 dimerization [2]. VEGF causes the release of activated VEGFR2 from lipid rafts, freeing it for internalization through clathrin-mediated endocytosis [30]. The internalization of Nrp1 has been shown to comprise two routes in ECs—stimulation with VEGF leading to endocytosis of Nrp1 via the clathrin-dependent pathway, whereas stimulation with semaphorin 3C leading to internalization via lipid rafts [31]. We find that raised expression of raftlin stabilizes the surface pool of Nrp1 and inhibits the endocytosis of activated VEGFR2 (Fig. 4). Conversely, depletion of the normal cellular pool of raftlin results in a significant loss of surface Nrp1 and an increase in endocytosis of activated VEGFR2 (Fig. 4). These findings support a model whereby raftlin stabilizes Nrp1 at the cell surface by anchoring it to lipid rafts. These raftlin–Nrp1 complexes then capture activated VEGFR2 and block its release from rafts, inhibiting its subsequent endocytosis through clathrin-dependent mechanisms. By increasing the pool of surface Nrp1 available for interaction with activated VEGFR2, raftlin would also support the recycling of internalized receptor [15] and we consequently see accumulation of VEGFR2 in the late endosomal compartment in ECs depleted of raftlin (Fig. 4d).

Regulation of the kinetics and routes of receptor trafficking is an important part of shaping the signaling output [18]. We find that raftlin has no effect on activation of the Erk1/2 pathway (Fig. 5), which controls EC proliferation in response to VEGFR2 activation [7]. We find that raftlin acts to suppress activation of p38, however (Fig. 5), which is required for the chemotactic response of ECs to VEGF [28]. Both raftlin and Nrp1 suppress the activation of FAK (as judged by phosphorylation of Tyr397), and also suppress the phosphorylation of Tyr925 (Fig. 5, S3), which is linked to increased cell motility [29]. Suppression by raftlin of Tyr925 phosphorylation is far greater than by Nrp1, suggesting that the dominant effect is supplied by raftlin. During angiogenesis, stalk cells at the base of the angiogenic sprout undergo proliferation in response to VEGF but do not show chemotactic motility. Conversely, tip cells do not proliferate but sense the chemotactic VEGF gradient [32, 33]. These coordinated and reciprocal responses are critical for the integrity and collective migration of the chord. Nrp1 is required for chemotactic sensing of VEGF by the tip cell [34], supporting its pro-migratory role in ECs. It will be important to examine the relative expression of raftlin in stalk and tip cells during angiogenesis to determine if this contributes to this differential response to VEGFR2 activation in the two cell subtypes.

The identification of raftlin as a novel component of the activated VEGFR2 signaling complex highlights that there are still important parts of the molecular mechanisms of VEGFR2 signaling to be identified. It is likely that the VEGFR2 proteome presented here contains further novel components of this network and we are currently working to validate additional new partners. It is clear that normal cellular levels of raftlin make a direct and significant contribution to the signaling output of VEGFR2 activation in ECs. One important remaining question is how raftlin itself may be regulated. Recent studies have shown that raftlin is highly upregulated in the serum of septic patients, with evidence that this derives from increased expression in ECs [35]. It will be important to determine the mechanisms controlling the expression of raftlin in ECs, and how responses to physiological and pathological conditions alter raftlin expression to regulate the cellular response to VEGFR2 activation.

## Electronic supplementary material

Below is the link to the electronic supplementary material.

Supplementary file1 (PDF 1115 kb)

Supplementary file2 (PDF 25417 kb)

Supplementary file3 (PDF 23 kb)

Supplementary file4 (XLS 1950 kb)
