# Supplementary material for: Raftlin is recruited by neuropilin-1 to the activated VEGFR2 complex to control proangiogenic signaling
Source: Angiogenesis. 2020 Apr 9;23(3):371–83. doi: 10.1007/s10456-020-09715-z (PMC7311514; doi:10.1007/s10456-020-09715-z)
Supplement: Supplementary file 1 — Supplementary file1 (PDF 1115 kb) [file 10456_2020_9715_MOESM1_ESM.pdf]

Figure S1

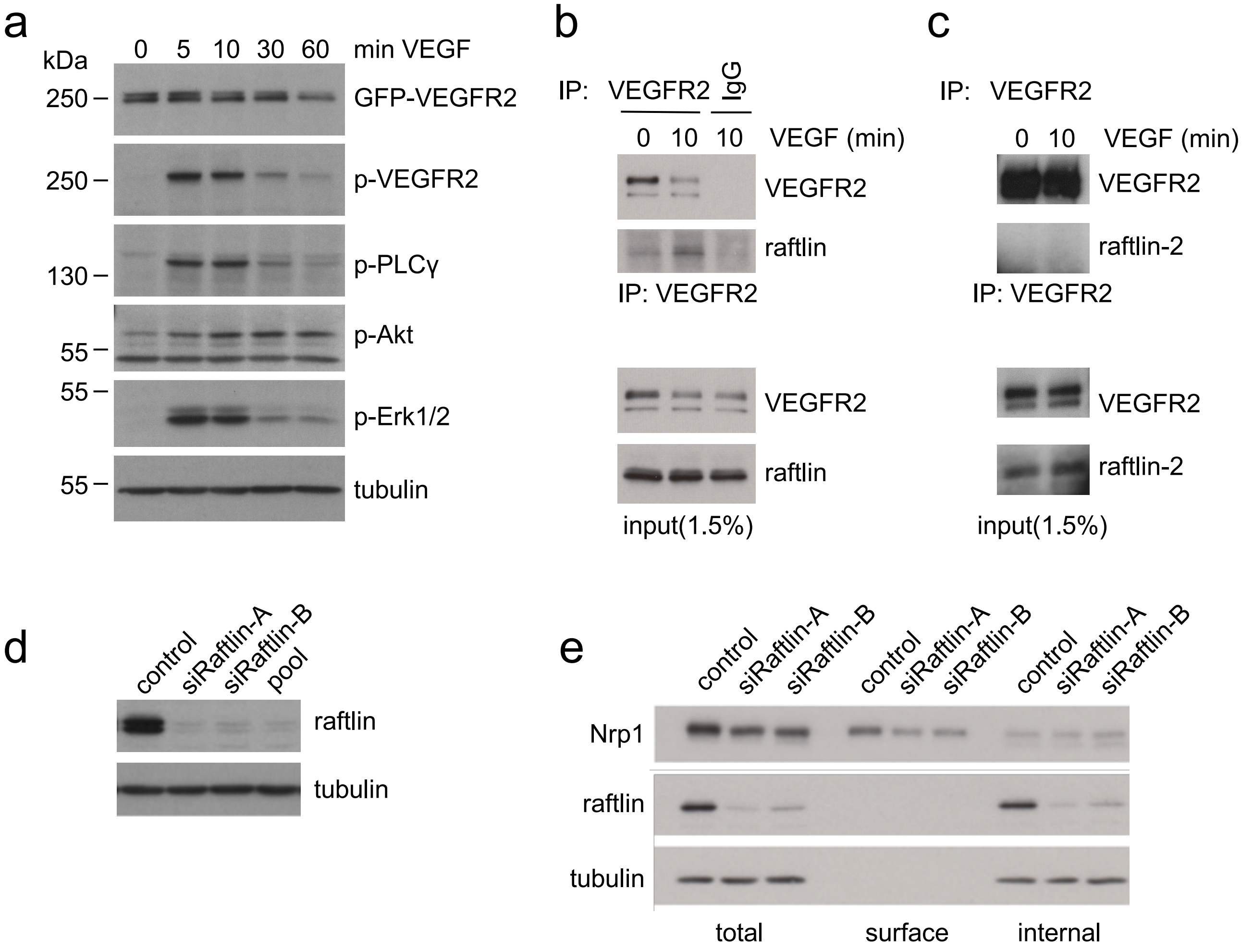

Fig. S1. Raftlin is recruited to activated VEGFR2. **a** CiGEnC expressing GFP-VEGFR2 were stimulated with 40ng/ml VEGF over a 60min time course. The phosphorylation of downstream signaling partners was detected by western blotting. GFP-VEGFR2 was detected at its predicted molecular weight and was phosphorylated at Tyr-1175 on VEGF stimulation. Other downstream targets were phosphorylated as expected. **b** ECs were stimulated ± 40ng/ml VEGF for 10min prior to harvesting and immunoprecipitation of endogenous VEGFR2. A small amount of endogenous raftlin was associated with the receptor in unstimulated cells, and this interaction increased significantly on VEGF stimulation. Control immunoprecipitation was with a non-specific IgG. **c** The experiment was repeated, and samples probed for raftlin-2. We did not detect raftlin-2 in these samples of purified VEGFR2 complex. **d** ECs were transfected with control siRNA, two independent raftlin siRNAs, or a pool of both. Effective silencing of endogenous raftlin was seen with both siRNAs. **e** HUVEC were transfected with raftlin siRNA or control. Surface proteins were biotinylated on ice and isolated using streptavidin beads. The panel shows a representative western blot. Silencing of raftlin caused a loss of surface Nrp1. Quantification is shown in Fig. 3d.
