# Supplementary material for: Raftlin is recruited by neuropilin-1 to the activated VEGFR2 complex to control proangiogenic signaling
Source: Angiogenesis. 2020 Apr 9;23(3):371–83. doi: 10.1007/s10456-020-09715-z (PMC7311514; doi:10.1007/s10456-020-09715-z)
Supplement: Supplementary file 2 — Supplementary file2 (PDF 25417 kb) [file 10456_2020_9715_MOESM2_ESM.pdf]

Figure S2

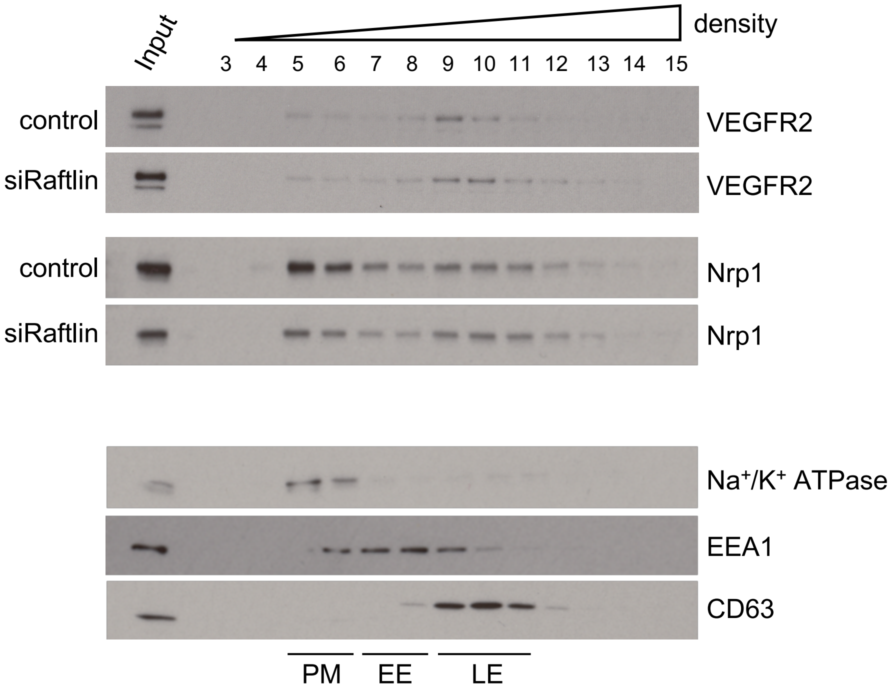

Fig. S2. Raftlin controls the internalization of VEGFR2. HUVEC were treated with raftlin siRNA or control and then stimulated with 40ng/ml VEGF for 30min. HUVEC were mechanically broken and cellular membranes purified by density centrifugation. The intracellular distributions of Nrp1 and VEGFR2 were determined by western blotting of gradient fractions. The Figure shows a representative blot and full Quantification is shown in Figure 4F. The use of markers of cellular compartments revealed the positions of the plasma membrane (PM), early endosome (EE) and late endosome (LE) fractions on the gradient. Silencing of raftlin caused a loss of surface Nrp1 and an increase of VEGFR2 in late endosomal fractions.
