# Supplementary material for: Raftlin is recruited by neuropilin-1 to the activated VEGFR2 complex to control proangiogenic signaling
Source: Angiogenesis. 2020 Apr 9;23(3):371–83. doi: 10.1007/s10456-020-09715-z (PMC7311514; doi:10.1007/s10456-020-09715-z)
Supplement: Supplementary file 3 — Supplementary file3 (PDF 23 kb) [file 10456_2020_9715_MOESM3_ESM.pdf]

Figure S3

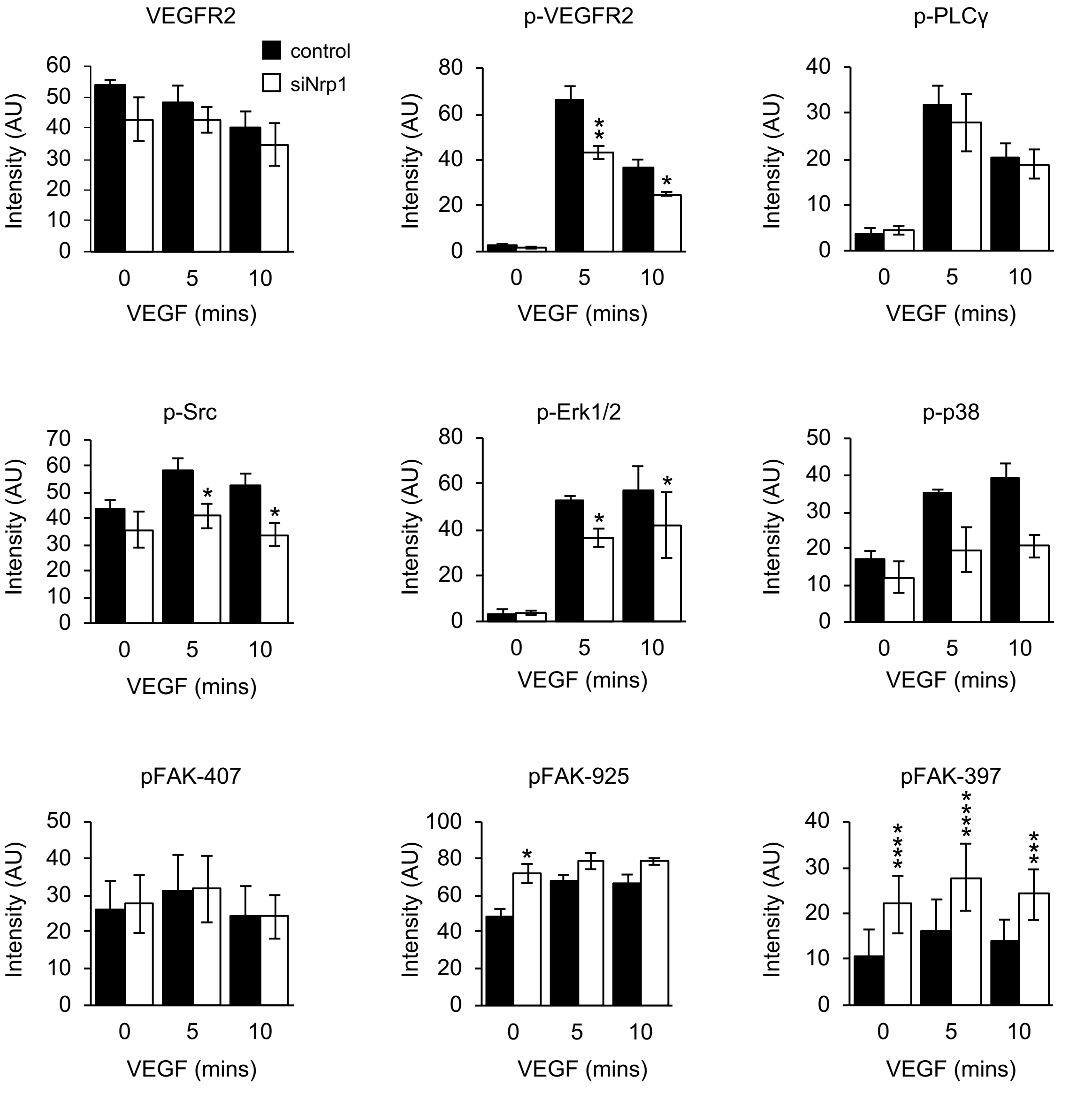

Fig. S3. Effects of Nrp1 silencing on VEGFR2 signaling. HUVEC were transfected with Nrp1 siRNA or control and then stimulated with 40ng/ml VEGF over a time course of 30min. The activation of key downstream VEGFR2 signaling partners was quantified by western blotting and densitometry. Depletion of Nrp1 decreased VEGFR2 activation, and also decreased the activation of Src, Erk1/2 and p38. Phosphorylation of FAK at Y397 was significantly increased. Data are mean ± SEM (n=3).
